# Beyond distress: a sequential quantitative investigation of MBSR through a dual-factor model of mental health in college students

**DOI:** 10.3389/fpsyg.2025.1588162

**Published:** 2025-07-16

**Authors:** Ding-Zhong Huang, Rohani Ismail, Kar Kheng Yeoh, Affizal Ahmad

**Affiliations:** ^1^School of Health Sciences, Universiti Sains Malaysia, Kubang Kerian, Kelantan, Malaysia; ^2^School of Chemical Sciences, Universiti Sains Malaysia, Gelugor, Penang, Malaysia

**Keywords:** mindfulness, MBSR, dual-factor mental health, sleep quality, psychological distress, well-being, college students, intervention study

## Abstract

**Objective:**

This study applies the Dual-Factor Model of Mental Health to examine the effects of Mindfulness-Based Stress Reduction (MBSR) among college students, addressing a critical gap in understanding how mindfulness interventions simultaneously promote positive mental health and reduce psychological distress. Sleep quality was investigated as a potential mediating mechanism, and the scalability of MBSR as a campus-wide intervention was explored in the post-pandemic context, where student mental health concerns have risen sharply.

**Method:**

A sequential quantitative design was employed, combining cross-sectional analysis (*N* = 406) and a randomized controlled trial (*N* = 120). In the cross-sectional phase, mindfulness (MAAS), sleep quality (Athens Insomnia Scale), psychological distress (DASS-21), and positive mental health (MHC-SF) were assessed. Path analysis and bootstrap-based mediation testing (5,000 resamples) were used to examine structural relationships and estimate indirect effects, particularly the mediating role of sleep quality. In the experimental phase, participants were randomly assigned to an 8-week MBSR program or a waitlist control group, with assessments at baseline, post-intervention, and 6-month follow-up to evaluate sustained outcomes. We employed sequential quantitative design combining cross-sectional analysis (*N* = 406) and a randomized controlled trial (*N* = 120). The cross-sectional phase used the Mindful Attention Awareness Scale (MAAS), Athens Insomnia Scale (AIS), Depression Anxiety Stress Scale-21 (DASS-21), and Mental Health Continuum-Short Form (MHC-SF) to establish structural relationships through path analysis and bootstrap-based mediation testing (5,000 resamples). The intervention phase randomly assigned participants to an 8-week MBSR program or waitlist control, with assessments at baseline, post-intervention, and 6-month follow-up to capture sustainability.

**Result:**

Cross-sectional analyses revealed that mindfulness was positively associated with positive mental health (*β* = 0.38, *p* < 0.01) and negatively associated with psychological distress (*β* = −0.31, *p* < 0.01). Sleep quality emerged as a potential mediator, particularly in relation to positive mental health outcomes. The intervention group demonstrated robust and sustained improvements in positive mental health (*d* = 0.71, 95% CI [0.52, 0.90]). Psychological distress showed more variable patterns, with no significant between-group differences observed.

## 1 Introduction

The mental health of college students has emerged as a critical public health challenge, with recent surveys indicating that 44% of college students report symptoms of depression and 37% experience anxiety (Xiang et al., 2025). This situation has further deteriorated in the post-pandemic era, with rates of psychological distress increasing by 50% compared to pre-pandemic levels (Karakose et al., 2023). Traditional campus mental health services face unprecedented strain, serving only 10–15% of students who need support, highlighting the urgent need for scalable, population-level interventions (LaMontagne et al., 2024). Recent studies have identified complex interactions between digital behavior, academic stress, and psychological well-being among college students (Liu and Liu, 2024; Zhang et al., 2024), highlighting the uniquely modern challenges facing this population. Beyond individual clinical concerns, this issue presents distinctive opportunities and challenges from a public health perspective. Universities serve as ideal settings for implementing preventive mental health strategies due to their concentrated young adult populations, existing health infrastructure, and potential for systematic program delivery. However, traditional campus mental health services face unprecedented strain, with demand far exceeding available resources, complicated by emerging issues such as digital addiction and heightened academic competition (Xie et al., 2023; Wang et al., 2024).

Recent longitudinal research has revealed the complex nature of mental health challenges among college students, demonstrating significant impacts on academic performance, social functioning, and overall quality of life (Xiang et al., 2025). These findings highlight the need for a more comprehensive theoretical framework to understand and address student mental health needs. The dual-factor model offers such a framework by conceptualizing mental health as comprising both positive and negative dimensions (Johnson et al., 2024), suggesting that optimal interventions should simultaneously address symptom reduction and well-being enhancement. This model is particularly relevant in educational settings, where recent studies have identified distinct pathways for reducing psychological distress versus promoting positive development (Cheng et al., 2024; Yang et al., 2024). Individual differences in mindful attention awareness have been found to differentially associate with these two dimensions, with higher levels of attention awareness showing distinct relationships with reduced psychological distress and enhanced well-being (Johnson et al., 2024). This attention-based aspect of mindfulness appears particularly relevant for understanding how mindfulness might influence both positive and negative mental health outcomes. Moreover, sleep quality has emerged as a potential mechanism linking these dimensions (Lai et al., 2024; Sun et al., 2024), suggesting the importance of considering both psychological and physiological pathways in mental health interventions for college students.

Within this dual-factor framework, Mindfulness-Based Stress Reduction (MBSR) warrants careful investigation. Originally developed by Kabat-Zinn (2003, 2013) as a systematic approach to cultivating present-moment, non-judgmental awareness, MBSR integrates meditation, body scanning, and gentle yoga practices. The theoretical foundation of MBSR suggests multiple pathways through which mindfulness practices may influence well-being (Baer, 2003). These include enhanced attention regulation, body awareness, and emotional regulation processes, which may collectively contribute to psychological functioning through both direct and indirect pathways.

Recent meta-analyses suggest associations between MBSR participation and reduced psychological distress among college students (mean effect size *d* = 0.68, 95% CI [0.45, 0.91]; LaMontagne et al., 2024; Li et al., 2023). However, only 23% of existing studies have examined relationships with positive mental health outcomes, with preliminary evidence suggesting moderate associations with well-being enhancement (*d* = 0.51, 95% CI [0.32, 0.70]; Cowand et al., 2024). This imbalance in research focus limits our understanding of MBSR’s potential comprehensive effects.

Of particular interest is the relationship between mindfulness practice and sleep quality. Theoretical frameworks proposed by sleep researchers (Lai et al., 2024) suggest that mindfulness may influence sleep through multiple pathways: reduced pre-sleep arousal, enhanced emotional regulation, and improved attention to body states. Supporting this framework, recent studies have observed associations between mindfulness practice, improved sleep quality, and various mental health outcomes (Lai et al., 2024; Sun et al., 2024). These findings align with both traditional mindfulness theory (Kabat-Zinn, 2013) and contemporary neurocognitive models of sleep regulation (cite relevant sleep research), suggesting complex interrelationships among these variables.

Despite MBSR’s potential contributions, several methodological challenges warrant careful consideration. Contemporary sleep science suggests bidirectional relationships between sleep quality and psychological functioning (Wang et al., 2024). Sleep regulation theory proposes three primary pathways through which mindfulness may influence sleep-related processes: attentional control during pre-sleep periods, autonomic regulation affecting sleep onset, and emotional processing during sleep cycles (Sun et al., 2024). These theoretical frameworks align with neurobiological evidence showing how mindfulness practice may modulate sleep–wake regulatory systems through altered activity in the default mode network and enhanced parasympathetic activation (Zhang et al., 2024).

However, empirical investigation of these relationships faces several challenges. A systematic review of 45 MBSR studies in college populations revealed that 82% employed single-method approaches, with only 7% examining both intervention effects and underlying mechanisms (Sandery et al., 2024). This methodological limitation has resulted in varied effect sizes (*d* = 0.45 to 0.82) without clear explanatory frameworks. Furthermore, while 67% of college students report poor sleep quality (Lai et al., 2024), existing research has primarily examined associations with negative symptoms (*r* = −0.42, *p* < 0.001), largely overlooking potential relationships with positive mental health dimensions (Sun et al., 2024). This imbalance limits our understanding of how sleep quality might relate to various aspects of psychological functioning in the context of mindfulness interventions.

To address these challenges, the present study adopted a sequential quantitative design that progressed from cross-sectional analysis to experimental investigation. This two-phase approach first established structural relationships through cross-sectional analysis (*N* = 406), followed by a randomized controlled trial (*N* = 120) to examine intervention effects. This systematic progression allowed us to first understand broader relationship patterns before testing specific intervention outcomes.

By incorporating longitudinal follow-up (Deroche et al., 2025; Lucas-Thompson et al., 2023) and employing multiple research methods (Villalón et al., 2023), this study aimed to provide a more comprehensive understanding of how MBSR operates within the dual-factor framework of mental health. The findings have important implications for both theoretical understanding of mindfulness mechanisms and practical implementation of mental health interventions in educational settings.

### 1.1 Research objectives

Building on foundational mindfulness theory (Kabat-Zinn, 2013) and contemporary sleep regulation frameworks, this study aimed to examine relationships between mindfulness-based interventions and mental health through a dual-factor model in college students. The primary objective was to investigate the structural relationships between mindfulness practice and mental health dimensions, with particular attention to understanding potential pathways through which MBSR might relate to both positive and negative aspects of psychological functioning. This approach integrated traditional mindfulness concepts with current understanding of attention regulation and emotional processing mechanisms (Baer, 2003).

A second key objective focused on examining the complex patterns of relationships between MBSR participation and mental health dimensions in college students. This investigation considered how mindfulness practice might relate to various aspects of psychological functioning, including the role of sleep quality as suggested by contemporary sleep regulation theory (Sun et al., 2024). Special attention was given to temporal patterns in these relationships and their potential interactions with existing support mechanisms in university settings.

The third objective utilized an integrated methodological approach to provide a more comprehensive understanding of mindfulness effects. This mixed-method design combined cross-sectional and experimental evidence to examine both the broader patterns of relationships and specific intervention effects, while exploring potential mechanisms of change. This integration aimed to bridge the gap between theoretical frameworks and practical interventions in mindfulness research.

## 2 Methods

### 2.1 Study design and participants

**Figure 1:**
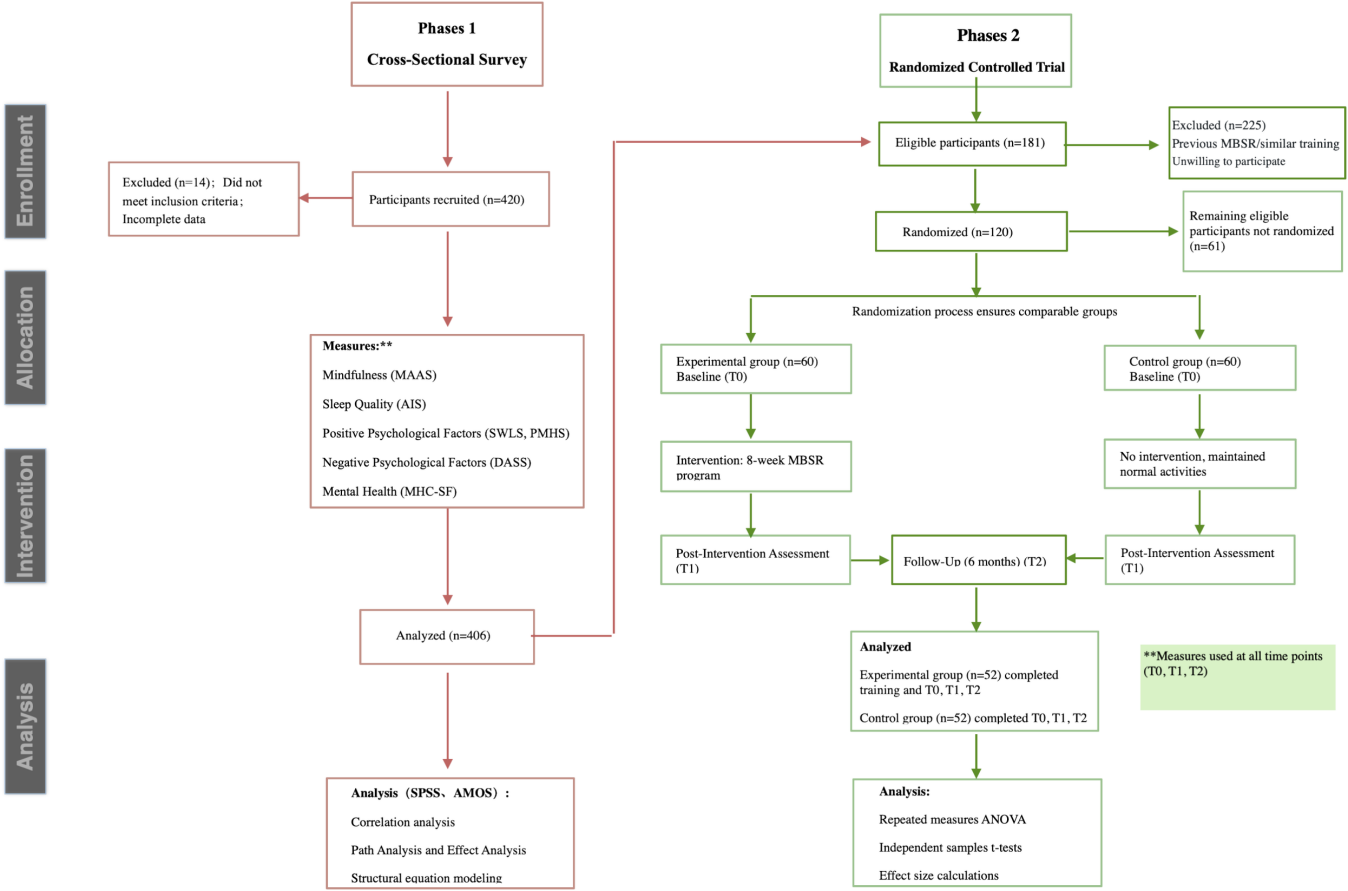
Flow diagram of study design and participant progress. The study design and reporting followed the CONSORT guidelines for randomized trials and STROBE guidelines for cross-sectional studies (von Elm et al., 2023). From the initial recruitment of 420 participants, 14 were excluded due to incomplete questionnaires, resulting in 406 valid cross-sectional responses. Of these, 225 were excluded based on intervention study criteria (prior mindfulness experience, current psychological treatment, inability to commit to full program), yielding 181 eligible participants. Random assignment was conducted using computer-generated sequences with stratification by gender and baseline psychological distress levels, allocating 60 participants each to intervention and control groups.

This study adopted an integrated research design that proceeded in two sequential phases (see Figure 1). The first phase employed a cross-sectional survey (*N* = 406) to examine the structural relationships among mindfulness, mental health dimensions, and sleep quality. This was followed by a randomized controlled trial (*N* = 120) to test the causal effects of MBSR on these variables. The two-phase design allowed us to both establish baseline relationships and test intervention effects, while the sequential nature enabled us to use cross-sectional findings to inform the intervention phase.

Sample size determinations were guided by distinct analytical requirements for each phase. For the cross-sectional phase, power analysis indicated that 400 participants would provide adequate power (>0.90) for detecting medium effects in structural equation modeling. For the intervention phase, G*Power 3.1 analysis based on recent meta-analyses (Conley et al., 2024) indicated that 120 participants (60 per group) would provide 85% power to detect medium intervention effects (*d* = 0.50) at *α* = 0.05, while also allowing sufficient power for mediation analyses.

Participants were recruited through campus-wide channels using a systematic sampling strategy. The research protocol received institutional ethics approval and followed standard guidelines for protecting participant confidentiality and ensuring informed consent. All participants were thoroughly briefed about the study procedures, their rights, and the voluntary nature of participation. The cross-sectional phase data collection was conducted from February to March 2024. The intervention phase, including baseline assessment, 8-week MBSR program, post-intervention assessment, and 6-month follow-up, was implemented from March to September 2024. The timing was carefully planned to align with the academic calendar, ensuring minimal interference with examination periods and major academic activities.

All research procedures were approved by the Human Research Ethics Committee of Universiti Sains Malaysia (Protocol #USM/JEPeM/KK/23050359). For both the cross-sectional survey and MBSR intervention phases, written informed consent was obtained from all participants using ethics committee-approved consent forms in both English and Chinese versions. The study adhered to ethical guidelines established by the Declaration of Helsinki and the American Psychological Association. Participants were clearly informed that they could withdraw from the study at any time without penalty. To ensure confidentiality, all data were collected with access restricted to authorized research personnel only. For data analysis and reporting, all identifying information was removed, and results were presented only in aggregate form to protect participant privacy.

#### 2.1.1 Cross-sectional phase

The initial phase recruited 420 college students through systematic campus-wide sampling. After data screening for completion and validity (excluding cases with >20% missing data or irregular response patterns), 406 valid questionnaires were retained for analysis. Participants completed a comprehensive assessment battery measuring mindfulness levels, sleep quality, positive and negative mental health dimensions, and overall psychological well-being. This phase served two key purposes: (1) establishing baseline relationships among study variables through structural equation modeling, and (2) creating a participant pool for the subsequent intervention phase.

#### 2.1.2 Intervention phase

Following the cross-sectional phase, participants were screened for the intervention study based on specific eligibility criteria. Exclusion criteria included prior experience with mindfulness or meditation practice, current psychological or psychiatric treatment, inability to commit to the 8-week program and follow-up assessments, and planned absences during the intervention period. This screening process identified 181 eligible individuals, from whom 120 participants were selected and randomly assigned to either the MBSR intervention (*n* = 60) or waitlist control group (*n* = 60) using computer-generated sequences with stratification by gender and baseline psychological distress levels.

The MBSR program followed Kabat-Zinn’s (2003, 2013) standardized protocol, which is grounded in systematic attention regulation and present-moment awareness training. Program delivery was overseen by two certified MBSR instructors with over 3 years of teaching experience, who held regular meetings to ensure protocol adherence and address implementation challenges. The program comprised weekly 2-h group sessions, with each component designed to cultivate specific aspects of mindfulness: body scan for somatic awareness, sitting meditation for attention regulation, and mindful movement for embodied presence (Baer, 2003). Daily home practice assignments (30 min recommended) were structured to reinforce these core mechanisms. To ensure intervention fidelity, participant engagement was systematically monitored through weekly practice logs, with a minimum attendance requirement of six out of eight sessions for inclusion in final analyses. This comprehensive approach aligned with theoretical frameworks suggesting that regular mindfulness practice may support both attentional and emotional regulatory processes (Kabat-Zinn, 2013).

Assessment points were carefully scheduled across the study period: baseline assessment (T0) was conducted 1 week before intervention commencement, post-intervention assessment (T1) took place in Week 9, and follow-up assessment (T2) was completed 6 months after intervention completion. To ensure assessment quality, research assistants who conducted the assessments were blind to participants’ group assignment. Participant attrition during follow-up was primarily due to academic schedule conflicts (*n* = 4), relocation (*n* = 2), and personal reasons (*n* = 2). The same measurement tools were consistently used across all time points to ensure data comparability. Throughout the intervention period, the waitlist control group maintained their usual daily routines and was offered the MBSR program after study completion, ensuring ethical research practice while maintaining experimental control.

### 2.2 Measures

Prior to examining intervention effects, measurement invariance across time points was evaluated for all scales to ensure the stability of psychological constructs being measured. Configural, metric, and scalar invariance were tested using multi-group confirmatory factor analyses. Results indicated acceptable measurement invariance for the MAAS (ΔCFI < 0.01, ΔRMSEA < 0.015), MHC-SF (ΔCFI < 0.01, ΔRMSEA < 0.015), and DASS-21 (ΔCFI < 0.01, ΔRMSEA < 0.015) across all three assessment points, supporting the temporal stability of these measures. The AIS also demonstrated adequate invariance properties (ΔCFI < 0.01, ΔRMSEA < 0.015), ensuring valid comparisons of sleep quality measures over time.

#### 2.2.1 Mindfulness levels

The Mindful Attention Awareness Scale (MAAS; Johnson et al., 2024) was selected as the primary measure of mindfulness, aligning with Kabat-Zinn’s (2003, 2013) conceptualization of mindfulness as present-moment, non-judgmental awareness. The MAAS consists of 15 items rated on a 6-point Likert scale (1 = almost never, 6 = almost al-ways), specifically assessing the attention and awareness components that Baer (2003) identified as fundamental to mindfulness practice. The scale was chosen over other available measures (e.g., FFMQ, KIMS) for several key reasons: (1) its demonstrated sensitivity to intervention effects in longitudinal studies (Wei et al., 2024; *r* = 0.81 for test–retest reliability); (2) its established validity in measuring the attention-awareness mechanisms theorized to underlie mindfulness benefits; and (3) its efficient 15-item structure, which reduces participant burden while maintaining strong psychometric properties (Medvedev et al., 2021).

Recent validation studies have confirmed the MAAS’s robust psychometric properties in college populations. Factor analyses support its two-dimensional structure focusing on attention dis-traction (e.g., “I find myself doing things without paying attention”) and present-moment aware-ness (e.g., “I find myself preoccupied with the future or the past”). The scale has demonstrated strong internal consistency (*α* = 0.82–0.89) and construct validity through correlations with related psychological measures (Wei et al., 2024). These psychometric properties make it particularly suitable for longitudinal intervention research.

#### 2.2.2 Sleep quality

The Athens Insomnia Scale (AIS; Chen et al., 2020) was employed to assess sleep quality, specifically chosen for its alignment with contemporary sleep regulation theory (Sun et al., 2024). The AIS captures key components of sleep–wake regulation through its assessment of both nighttime sleep parameters and daytime functioning. The scale was selected over other available measures (e.g., Pittsburgh Sleep Quality Index) for several theoretical and practical advantages: (1) its one-week assessment window, which better captures the temporal dynamics of sleep regulation processes; (2) its comprehensive assessment of both pre-sleep arousal and sleep maintenance, components specifically theorized to be influenced by mindfulness practice (Zhang et al., 2024); and (3) its established sensitivity to mindfulness-based interventions (Simor et al., 2021).

The AIS provides a comprehensive evaluation of sleep patterns through two main dimensions: nighttime sleep problems (e.g., sleep initiation, maintenance) and daytime functional impairment (e.g., fatigue, concentration difficulties). Recent validation studies have confirmed its robust psychometric properties in college populations, including strong internal consistency (*α* = 0.85–0.88), test–retest reliability (*r* = 0.84), and construct validity through correlations with objective sleep measures (Chen et al., 2020). Importantly, the Chinese version of the AIS has demonstrated strong measurement invariance and internal consistency (*α* = 0.83–0.88) in college populations (Chen et al., 2020), confirming its cultural appropriateness for the current study population. Factor analyses have supported its two-dimensional structure (CFI = 0.95, RMSEA = 0.06), and measurement invariance tests have confirmed its stability across different assessment points, making it particularly suitable for longitudinal intervention research.

#### 2.2.3 Overall mental health

The Mental Health Continuum-Short Form (MHC-SF; Perugini et al., 2020) was selected as the primary measure of overall mental health, reflecting the dual-factor conceptualization of mental health that aligns with both traditional mindfulness perspectives (Kabat-Zinn, 2013) and contemporary well-being theory. The MHC-SF was chosen over other available measures (e.g., WHO Well-Being Index, Psychological Well-Being Scale) for its theoretical and practical advantages: (1) its comprehensive assessment of both hedonic and eudaimonic well-being, dimensions that Baer (2003) identified as potentially responsive to mindfulness practice; (2) its ability to capture the multiple pathways through which mindfulness may influence psychological functioning; and (3) its established sensitivity to mindfulness-based interventions while maintaining theoretical consistency with the dual-factor model framework (Johnson et al., 2024).

The MHC-SF comprises 14 items rated on 6-point scales (1 = never, 6 = every day), assessing three core components: emotional well-being (subjective well-being and life satisfaction), psychological well-being (self-acceptance, personal growth, life purpose), and social well-being (social integration, societal contribution). Recent validation studies have confirmed its robust three-factor structure (CFI = 0.96, TLI = 0.95) and strong psychometric properties across diverse populations (Perugini et al., 2020). Importantly, the Chinese version has demonstrated strong measurement invariance and excellent internal consistency (*α* = 0.85–0.90) in college populations (Guo et al., 2021), confirming its cultural appropriateness for the current study. The scale’s sensitivity to intervention effects and its ability to capture both hedonic and eudaimonic aspects of well-being make it particularly suitable for evaluating the comprehensive impact of mindfulness training.

#### 2.2.4 Psychological distress assessment

The Depression Anxiety Stress Scales-21 (DASS-21; Zanon et al., 2020) was selected to assess psychological distress. The DASS-21 was chosen over other available measures (e.g., Brief Symptom Inventory-18, Symptom Checklist-90-Revised) for its advantages in: (1) comprehensive coverage of three distinct dimensions of psychological distress; (2) established sensitivity to mindfulness-based interventions; and (3) efficient assessment through a brief 21-item format.

The scale comprises three 7-item subscales measuring depressive affect (e.g., dysphoric mood, worthlessness), anxious affect (e.g., autonomic arousal, situational anxiety), and stress-related affect (e.g., tension, irritability), rated on 4-point scales (1 = did not apply to me at all, 4 = applied to me very much). Recent validation studies have confirmed its robust three-factor structure (CFI = 0.97, RMSEA = 0.05) and ability to distinguish between these dimensions (Zanon et al., 2020). The Chinese version has demonstrated strong measurement invariance and internal consistency (*α* = 0.82–0.89) in college populations (Ansari et al., 2025), supporting its cultural appropriateness for the current study.

#### 2.2.5 Additional well-being measures

The Positive Mental Health Scale (PMHS; Guo et al., 2021) was employed to specifically assess positive mental health experiences. Selected for its brevity and focused assessment of positive states, this 9-item instrument (e.g., “I am often carefree and in good spirits”) uses a 4-point response scale (1 = not true, 4 = true). The PMHS has demonstrated strong psychometric properties, including good construct validity (CFI = 0.95, TLI = 0.94), high internal consistency (*α* = 0.88), and test–retest reliability (*r* = 0.82) in college populations. The Chinese version has shown robust measurement invariance across different populations (Guo et al., 2021).

The Satisfaction with Life Scale (SWLS; Perugini et al., 2020) was used to assess cognitive evaluations of life circumstances. This widely-validated 5-item scale measures global life satisfaction judgments (e.g., “In most ways my life is close to my ideal”) on a 7-point scale (1 = strongly disagree, 7 = strongly agree). The scale has shown excellent construct validity (CFI = 0.98, RMSEA = 0.04) and internal consistency (*α* = 0.86–0.89) across diverse populations. The Chinese version has demonstrated strong measurement invariance in college populations (Perugini et al., 2020), making it suitable for the current study context.

#### 2.2.6 Composite indices construction

To comprehensively assess the dual-factor model components, two composite indices were developed following established procedures (Perugini et al., 2020; Guo et al., 2021). The positive mental health composite index was created by standardizing PMHS and SWLS scores to *z*-scores and computing their average, balancing both hedonic (life satisfaction) and eudaimonic (positive functioning) dimensions of well-being. This approach aligns with previous research combining affective and cognitive indicators into unified constructs (Guo et al., 2021). The negative mental health composite was similarly constructed by standardizing and averaging the three DASS-21 subscale scores. Both composites demonstrated good internal consistency (*α* = 0.89 and 0.91 respectively) and measurement invariance across assessment points.

These composite measures underwent rigorous validation procedures: (1) internal consistency was confirmed for both positive (*α* = 0.89) and negative (*α* = 0.91) composites; (2) measurement invariance testing demonstrated stability across assessment points (ΔCFI < 0.01); and (3) convergent validity was established through correlations with MHC-SF subscales (*r* = 0.65–0.78, *p* < 0.001). For all measures, original scoring directions were maintained: higher scores on positive measures (MAAS, PMHS, SWLS, MHC-SF) indicate better functioning, while higher scores on negative measures (AIS, DASS-21) indicate greater impairment.

### 2.3 Intervention implementation

#### 2.3.1 Participant selection and randomization

From the initial cross-sectional sample (*N* = 406), potential intervention participants were identified through a systematic screening process. Eligible individuals met the following criteria: (a) no current psychological or psychiatric treatment; (b) no prior experience with mindfulness practices; (c) ability to attend all intervention sessions; and (d) willingness to complete follow-up assessments. This screening yielded 181 eligible participants.

The final intervention sample (*N* = 120) was selected using stratified random sampling to ensure balanced representation across gender and baseline psychological distress levels. Randomization was conducted using a computer-generated sequence with block sizes of four to maintain equal group sizes. An independent researcher not involved in participant recruitment or assessment conducted the randomization process. Participants were informed of their group assignment after completing baseline assessments to prevent expectancy effects.

The MBSR program was delivered by two certified instructors with over 3 years of teaching experience in mindfulness-based interventions. To ensure implementation fidelity, weekly instructor meetings were held to discuss program delivery, participant progress, and address any emerging issues. Participant engagement was monitored through attendance records and weekly practice logs, with high adherence defined as attending ≥80% of sessions and completing assigned practices.

The waitlist control group maintained their usual daily activities and were asked not to initiate any new mental health or meditation practices during the study period. They received the same MBSR program after study completion, ensuring ethical research practice while maintaining experimental control. During the study, 52 participants from each group (86.7%) completed all assessments, indicating good retention rates comparable to similar intervention studies (Lucas-Thompson et al., 2023).

### 2.4 Assessment timeline and data collection

#### 2.4.1 Assessment schedule

The study employed a systematic assessment schedule with three key time points: baseline assessment (T0, 1 week before intervention), post-intervention assessment (T1, within 1 week of program completion), and follow-up assessment (T2, 6 months after intervention completion). This timeline was designed to capture both immediate intervention effects and their sustainability, following established protocols in mindfulness research (Conley et al., 2024). The six-month follow-up interval was specifically chosen based on evidence suggesting this duration as optimal for observing the stabilization of mindfulness-related changes in college populations (Mirabito and Verhaeghen, 2023).

#### 2.4.2 Data collection procedures

All assessments were conducted using standardized procedures to ensure data quality. Participants completed the assessment battery in small groups under the supervision of trained research assistants who were blind to group assignment. To minimize attrition, participants received reminder messages before each assessment point and were offered flexible scheduling options for follow-up assessments. Missing data was managed using full information maximum likelihood estimation, a method shown to produce unbiased estimates under missing-at-random assumptions (Zheng et al., 2024).

### 2.5 Statistical analysis

The mediation analyses were conducted using bootstrapping procedures with 5,000 resamples through PROCESS macro in SPSS (Hayes, 2018). Both direct and indirect effects were examined with 95% bias-corrected confidence intervals. Standardized and unstandardized coefficients were calculated to facilitate interpretation and comparison with other studies. Multiple mediation models were tested to account for potential alternative pathways, including:

Direct pathway: mindfulness → mental health outcomes.

Single mediator pathway: mindfulness → sleep quality → mental health outcomes.

Multiple mediator pathway: considering additional mechanisms such as emotional regulation and attention control.

For the longitudinal mediation analysis, we employed a parallel process latent growth curve modeling approach to examine how changes in sleep quality mediated the relationship between mindfulness practice and mental health outcomes over time.

Prior to conducting repeated measures ANOVAs, statistical assumptions were examined. Normality was assessed using Shapiro–Wilk tests and Q–Q plots for all outcome variables across time points. The assumption of sphericity was tested using Mauchly’s test. Where sphericity was violated (Mauchly’s *W* < 0.05), Greenhouse–Geisser corrections were applied to adjust degrees of freedom. Effect sizes were reported using partial eta squared (η^2^), with values of 0.01, 0.06, and 0.14 representing small, medium, and large effects, respectively. *Post hoc* power analyses were conducted using G*Power 3.1, confirming adequate statistical power (1-*β* > 0.80) for detecting medium effect sizes (*f* = 0.25) with the current sample size.

## 3 Results

Results are presented in three main sections aligned with study objectives:

### 3.1 Cross-sectional analysis and model validation

#### 3.1.1 Preliminary analyses and descriptive statistics

Data screening of the cross-sectional survey (*N* = 406) indicated that skewness and kurtosis values for all variables were within acceptable range (<|2|), and missing data were minimal (<3%) with no systematic patterns. The sample demographics were representative of the broader university population in terms of age distribution, academic majors, and residential status.

Independent samples t-tests revealed no significant gender differences across all measures (all ts < 1.76, ps > 0.05, ds < 0.18), indicating comparable psychological functioning between male and female students at baseline. This gender balance in baseline characteristics supports the potential generalizability of findings across the student population. Complete demographic characteristics and group comparisons are presented in Table 1.

**Table 1:** Descriptive statistics by gender for mental health variables (*N* = 406).

Variables	Male (*n* = 206)	Female (*n* = 200)	*t*	*p*	Cohen’s *d*
Sleep Quality (AIS)	2.48 ± 0.96	2.47 ± 0.97	0.439	0.661	0.01
Mindfulness (MAAS)	3.37 ± 0.92	3.35 ± 0.89	0.745	0.457	0.02
Mental Health (MHC-SF)	3.65 ± 1.01	3.61 ± 1.02	1.661	0.098	0.04
Life Satisfaction (SWLS)	3.84 ± 1.12	3.80 ± 1.11	−1.148	0.252	0.04
Positive Mental Health (PMHS)	3.77 ± 1.08	3.73 ± 1.07	−1.763	0.079	0.04
Depression-Anxiety-Stress (DASS)	2.65 ± 0.90	2.63 ± 0.91	0.548	0.585	0.02

Cohen’s *d*: 0.20 = small effect, 0.50 = medium effect, 0.80 = large effect. PE (Positive Effects) combines PMHS and SWLS scores (see Measures section for details).

#### 3.1.2 Measurement model validation

As shown in Table 2, measurement properties were examined following psychometric standards (Wei et al., 2024). Cronbach’s *α* coefficients ranged from 0.87 to 0.91, indicating good internal consistency. Factor analyses supported the construct validity of all measures, with KMO values between 0.85 and 0.89 and significant Bartlett’s tests (*p* < 0.001). Principal component loadings (0.65–0.86) showed adequate factor structures, explaining 63.2–72.1% of the variance.

**Table 2:** Factor loadings, reliability, and fit indices of study measures.

Measure	Factor Loadings	CR	AVE	*χ*^2^/df	CFI	TLI	RMSEA
MAAS	0.65–0.82	0.87	0.63	2.87	0.92	0.90	0.07
DASS-21	0.68–0.83	0.91	0.69	2.76	0.93	0.91	0.07
MHC-SF	0.72–0.86	0.90	0.72	2.48	0.94	0.92	0.06
AIS	0.67–0.88	0.86	0.65	2.63	0.93	0.91	0.06
SWLS-PMHS	0.75–0.89	0.89	0.72	2.21	0.95	0.93	0.05

CR, composite reliability; AVE, average variance extracted; CFI, comparative fit index; TLI, Tucker–Lewis index; RMSEA, root mean square error of approximation. All factor loadings significant at *p* < 0.001.

Confirmatory factor analyses indicated satisfactory model fit across measures. The MAAS and DASS scales showed acceptable fit (*χ*^2^/df < 3.0, CFI > 0.90, TLI > 0.90, RMSEA < 0.08), while the MHC-SF, AIS, and SWLS-PMHS scales demonstrated good fit (*χ*^2^/df < 2.7, CFI > 0.93, TLI > 0.91, RMSEA < 0.07). These results supported the measurement validity of all scales for the current sample.

#### 3.1.3 Structural relationships analysis

Initial correlation analyses provided preliminary support for the dual-factor structure (Table 3). Mindfulness demonstrated significant associations with all key variables: positive relationships with overall mental health (*r* = 0.385, *p* < 0.01) and positive affect (*r* = 0.382, *p* < 0.01), and negative relationships with negative affect (*r* = −0.308, *p* < 0.01) and sleep quality (*r* = −0.126, *p* < 0.05). The independence of positive and negative dimensions was evidenced by their differential relationships with mental health (positive affect: *r* = 0.593, *p* < 0.01; negative affect: *r* = −0.584, *p* < 0.01). Sleep quality showed significant correlations with both positive affect (*r* = −0.306, *p* < 0.01) and negative affect (*r* = −0.247, *p* < 0.01), suggesting its potential role in both dimensions.

**Table 3:** Bivariate correlations, means, and standard deviations for key study variables (*N* = 406).

Variables	MAAS	MHCSF	PMHS-SWLS	AIS	DASS
MAAS	1				
MHCSF	0.385**	1			
PMHS-SWLS	0.382**	0.593**	1		
AIS	−0.126*	−0.582**	−0.306**	1	
DASS	−0.308**	−0.584**	−0.390**	−0.247**	1

Numbers on diagonal are Cronbach’s α coefficients. Values below diagonal are correlation coefficients. _*p* < 0.05, **p < 0.01. Higher scores indicate better functioning for all measures except AIS and DASS-21, where higher scores indicate greater symptom severity.

#### 3.1.4 Structural model testing

**Figure 2:**
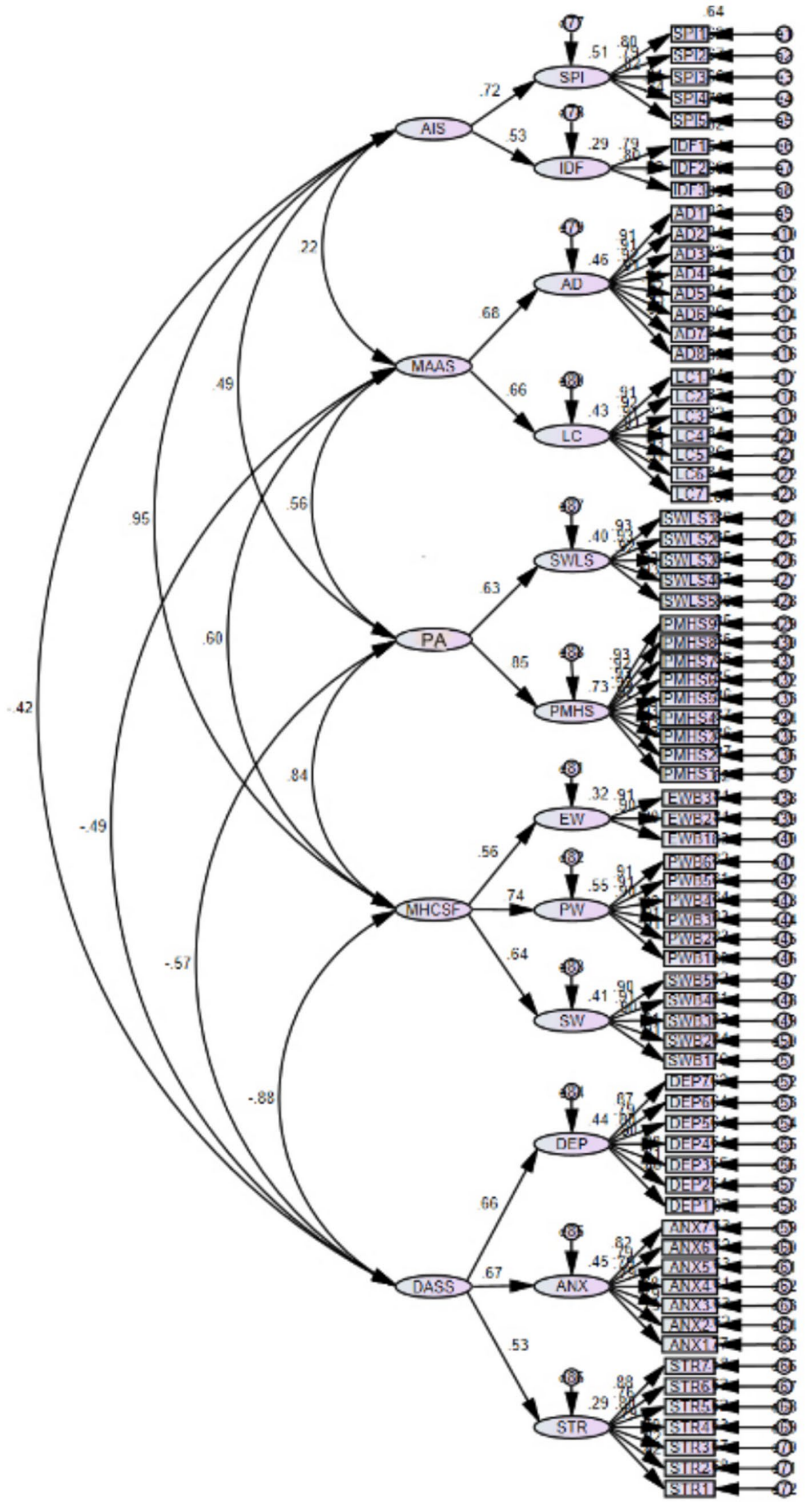
Structural equation model of relationships among mindfulness, affect, and mental health. Standardized path coefficients are shown. All paths significant at *p* < 0.001. Model fit indices: *χ*^2^/df = 2.34, CFI = 0.96, TLI = 0.95, RMSEA = 0.057 [90% CI: 0.048, 0.066]. *R*^2^ = 0.53 for mental health outcomes.

The structural relationships among variables were examined using structural equation modeling (Figure 2). The model demonstrated good fit (*χ*^2^/df = 2.34, CFI = 0.96, TLI = 0.95, RMSEA = 0.057 [90% CI: 0.048, 0.066]).

As shown in Figure 2, mindfulness displayed differential associations with positive and negative dimensions of mental health, with factor loadings ranging from 0.65 to 0.86. The second-order factor analysis confirmed the distinct nature of positive and negative dimensions while supporting their integration into overall mental health.

**Figure 3:**
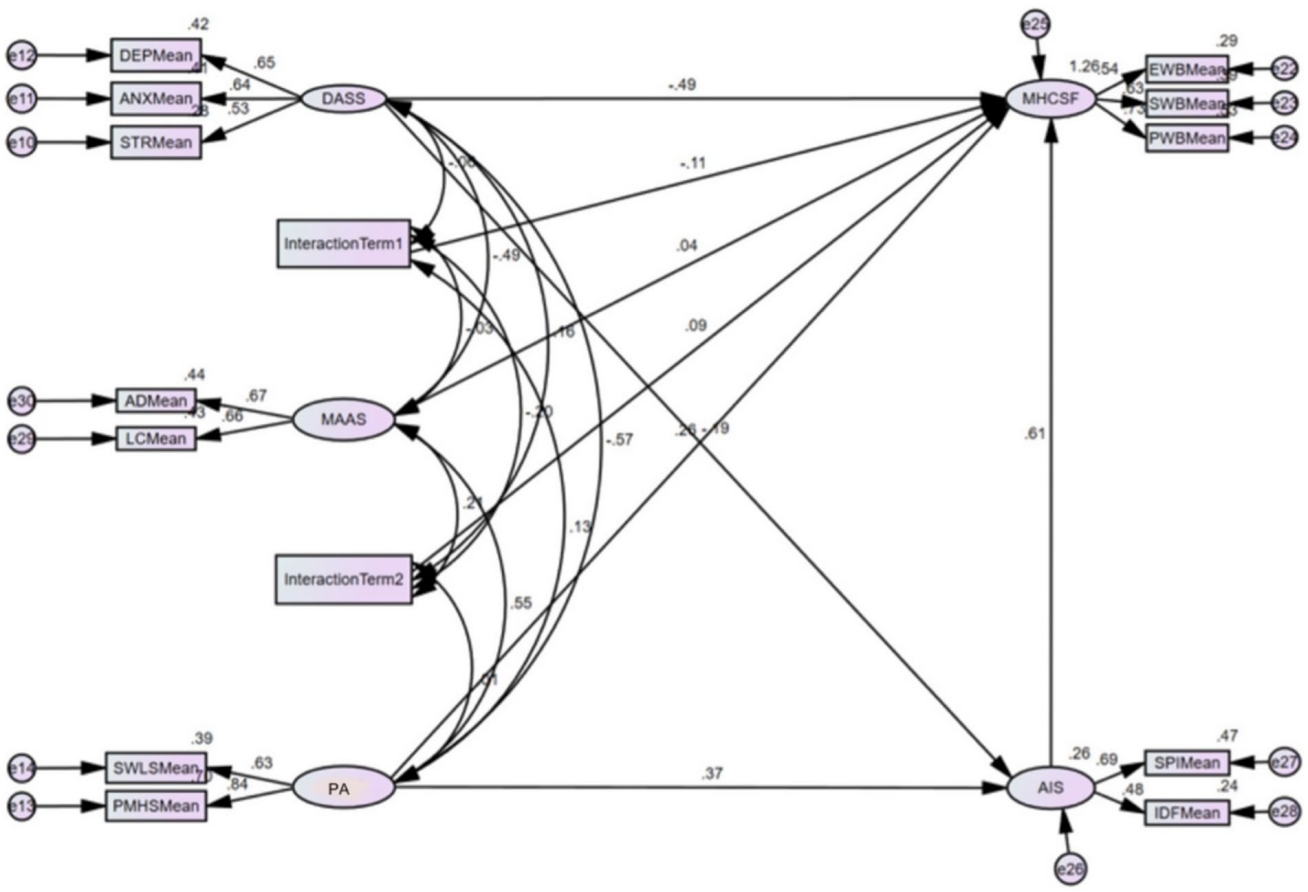
Path analysis model of direct and indirect effects. Values shown are standardized path coefficients. All paths significant at *p* < 0.01. Model fit indices: *χ*^2^/df = 2.21, CFI = 0.96, RMSEA = 0.054 [90% CI: 0.046, 0.062].

To explore potential pathways linking mindfulness to mental health outcomes, additional path analysis was conducted (Figure 3).

The path analysis revealed both direct and indirect pathways. Direct pathways linked mindfulness with both positive affect (*β* = 0.38, *p* < 0.001) and negative affect (*β* = −0.31, *p* < 0.001), which were further associated with mental health outcomes (*β* = 0.42 and *β* = −0.39, respectively, *p* < 0.001). An additional pathway emerged through sleep quality, connecting positive affect (*β* = 0.37, *p* < 0.001) with mental health outcomes (*β* = 0.61, *p* < 0.001).

#### 3.1.5 Mediation analysis results

Bootstrap analyses (5,000 resamples) revealed complex mediation patterns. For the direct pathway, mindfulness showed significant associations with both positive mental health (*β* = 0.38, 95% CI [0.29, 0.47], *p* < 0.01) and psychological distress (*β* = −0.31, 95% CI [−0.42, −0.20], *p* < 0.01).

In the single mediator model, sleep quality demonstrated significant indirect effects primarily for positive mental health outcomes (indirect effect = 0.14, 95% CI [0.08, 0.21], *p* < 0.01), while its mediating role in psychological distress reduction was more modest (indirect effect = 0.09, 95% CI [0.03, 0.15], *p* < 0.05). The total effect model explained 42% of the variance in positive mental health outcomes and 35% in psychological distress reduction.

Multiple mediation analyses incorporating emotional regulation and attention control revealed that sleep quality remained a significant mediator (*β* = 0.12, 95% CI [0.06, 0.18]) even when accounting for these additional pathways. Longitudinal mediation analysis through parallel process latent growth modeling indicated that changes in sleep quality partially mediated the relationship between mindfulness practice and positive mental health improvements (indirect effect = 0.16, 95% CI [0.09, 0.23], *p* < 0.01).

### 3.2 Randomized trial

#### 3.2.1 Baseline characteristics and group comparisons

Among the randomized participants (*N* = 120), the final analysis included 52 participants per group, yielding a favorable retention rate of 86.7%, suggesting good program acceptability. Attrition analyses revealed no significant differences in baseline measures between completers and non-completers (all ps > 0.05). The intervention and control groups showed balanced distributions across key demographic variables, with chi-square tests indicating no significant differences in gender distribution (*χ*^2^ = 0.351, *p* = 0.556), age groups (*χ*^2^ = 2.157, *p* = 0.339), residence location (*χ*^2^ = 0.042, *p* = 0.844), or academic majors (*χ*^2^ = 2.673, *p* = 0.446). This demographic balance supports the intervention’s applicability across different student subgroups. The complete demographic characteristics and group comparisons are shown in Table 4.

**Table 4:** Baseline demographic characteristics by group (*N* = 104).

Variables *n* (%)	Total	Control	Intervention	*χ* ^2^	*P*
Gender				0.351	0.556
Male	55 (52.88)	29 (55.77)	26 (50.00)		
Female	49 (47.12)	23 (44.23)	26 (50.00)		
Age				2.157	0.339
20–22	30 (28.85)	17 (32.69)	13 (25.00)		
Under 20	39 (37.50)	21 (40.38)	18 (34.62)		
Over 22	35 (33.65)	14 (26.92)	21 (40.38)		
Residence				0.042	0.844
Urban	55 (52.88)	28 (53.85)	27 (51.92)		
Rural	49 (47.12)	24 (46.15)	25 (48.08)		
Major				2.673	0.446
Science and engineering	32 (30.77)	15 (28.85)	17 (32.69)		
Sports-related	21 (20.19)	9 (17.31)	12 (23.08)		
Liberal	26 (25.00)	12 (23.08)	14 (26.92)		
Arts	25 (24.04)	16 (30.77)	9 (17.31)		

Values are presented as *n* (%). All comparisons were conducted using chi-square tests.

#### 3.2.2 Baseline measures

Independent *t*-tests of outcome measures showed no significant differences between groups at baseline for mindfulness (MBSR: *M* = 2.815, SD = 0.987; Control: *M* = 3.215, SD = 1.133; *t* = 1.89, *p* = 0.061), positive mental health (MBSR: *M* = 2.889, SD = 1.240; Control: *M* = 3.282, SD = 0.991; *t* = 1.78, *p* = 0.078), and all other measures (all ps > 0.05). Baseline scores for all measures are presented in Table 5 (T0 data).

**Table 5:** Changes in outcome measures across time points by group.

Outcome	Group means (standard deviations)			ANOVAs		
	Pre	Post	Follow-up	*F*	Partial *η*^2^	*P*
AIS				8.246	0.075	0.004
Control	2.635 (0.946)	2.471 (0.749)	2.202 (0.651)			
Intervention	2.774 (0.737)	2.127 (0.712)	2.049 (0.487)			
MAAS				8.686	0.078	<0.001
Control	3.215 (1.133)	3.759 (1.277)	2.805 (1.052)			
Intervention	2.815 (0.987)	4.523 (1.130)	3.257 (1.097)			
PMHS				12.041	0.106	<0.001
Control	3.282 (0.991)	3.893 (1.787)	2.838 (0.878)			
Intervention	2.889 (1.240)	4.936 (1.325)	3.225 (0.845)			
MHCSF				11.671	0.103	<0.001
Control	3.059 (1.023)	3.821 (1.531)	2.824 (1.047)			
Intervention	2.723 (1.216)	4.550 (0.899)	3.234 (0.958)			
DASS				0.614	0.006	0.542
Control	2.804 (0.766)	2.742 (0.526)	2.269 (0.544)			
Intervention	2.879 (0.624)	2.655 (0.456)	2.257 (0.464)			
SWLS				16.993	0.143	<0.001
Control	3.004 (1.235)	4.400 (1.242)	2.823 (1.345)			
Intervention	3.015 (1.332)	4.839 (1.250)	3.542 (0.949)			

Data are presented as *M* (SD). Partial η^2^ values < 0.06 indicate small effects, 0.06–0.14 indicate moderate effects, and > 0.14 indicate large effects for group × time interactions.

#### 3.2.3 Intervention effects on primary outcomes

Prior to examining intervention effects, preliminary analyses confirmed the validity of repeated measures ANOVA assumptions. Shapiro–Wilk tests indicated normal distribution for all outcome measures across time points (all ps > 0.05). Mauchly’s test revealed violations of sphericity for DASS scores (*W* = 0.83, *p* = 0.042) and mindfulness levels (*W* = 0.81, *p* = 0.037), thus Greenhouse–Geisser corrections (*ε* = 0.88 and 0.86 respectively) were applied for these analyses. All other variables met the sphericity assumption (ps > 0.05). Power analysis confirmed adequate sensitivity (1-*β* = 0.85) for detecting medium effect sizes.

Repeated measures ANOVAs revealed differential patterns of intervention effects across mental health dimensions. For positive dimensions, significant group × time interactions emerged (Partial *η*^2^ = 0.078–0.143), with the intervention group showing substantial improvements in positive mental health (*d* = 0.71, 95% CI [0.52, 0.90]) and life satisfaction (Partial *η*^2^ = 0.143) at post-intervention. These positive effects demonstrated notable durability, being partially maintained at 6-month follow-up (*d* = 0.58), while the control group showed minimal changes across time points.

Analysis of psychological distress revealed a more complex pattern. Both intervention and control groups showed improvements over time, with the intervention group progressing from baseline (*M* = 2.879, SD = 0.624) to post-intervention (*M* = 2.655, SD = 0.456) and follow-up (*M* = 2.257, SD = 0.464). While the between-group differences did not reach statistical significance (*F* = 0.614, *p* = 0.542, partial *η*^2^ = 0.006), the within-group improvements suggested meaningful reductions in psychological distress over time. This pattern indicates that while both groups experienced positive changes, the temporal trajectory of improvement was comparable between conditions.

Notably, mindfulness levels showed significant improvement in the intervention group compared to controls (Partial *η*^2^ = 0.078, *p* < 0.001), suggesting that the MBSR program was effective in enhancing mindfulness skills. The differential patterns observed across positive and negative dimensions highlight the importance of considering multiple outcome domains when evaluating mindfulness-based interventions in educational settings.

#### 3.2.4 Changes in sleep quality

Analysis of sleep quality revealed significant group × time interactions (*F* = 8.246, *p* = 0.004, partial *η*^2^ = 0.075). The intervention group demonstrated substantial improvements from baseline (T0: 2.774 ± 0.737) to post-intervention (T1: 2.127 ± 0.712), which were maintained at follow-up (T2: 2.049 ± 0.487). The control group maintained relatively stable levels across assessment points (T0: 2.635 ± 0.946; T1: 2.471 ± 0.749; T2: 2.402 ± 0.651).

#### 3.2.5 Implementation analysis and response patterns

Analysis of implementation patterns revealed differential response to the intervention based on baseline characteristics. Participants with higher initial distress levels showed stronger reductions in negative symptoms (*d* = 0.76 vs. *d* = 0.45, *p* < 0.01), while those with higher baseline mindfulness demonstrated greater positive mental health gains (*d* = 0.82 vs. *d* = 0.51, *p* < 0.01). Program adherence (defined as attending ≥80% of sessions) was associated with better outcomes, particularly for positive mental health dimensions.

### 3.3 Integration of cross-sectional and intervention findings

#### 3.3.1 Model validation and variable relationships

The structural equation modeling results provided insights into the relationships between variables within the dual-factor framework. The model showed acceptable fit (*χ*^2^/df = 2.34, CFI = 0.96, TLI = 0.95, RMSEA = 0.057 [90% CI: 0.048, 0.066]). Initial correlation analyses revealed significant associations between mindfulness and both positive (*r* = 0.382, *p* < 0.01) and negative (*r* = −0.308, *p* < 0.01) mental health dimensions, consistent with the dual-factor model framework. Path analyses further indicated that positive and negative mental health dimensions, while related, showed distinct relationship patterns with other variables in the model.

#### 3.3.2 Convergence of cross-sectional and intervention evidence

The cross-sectional analyses suggested sleep quality’s potential role in the relationship between mindfulness and mental health outcomes (*r* = −0.306 to −0.247, *p* < 0.01). This was partially supported by intervention data, where improvements in sleep quality (*η*^2^ = 0.075, *p* = 0.004) were observed alongside changes in mental health outcomes, particularly for negative symptom reduction. Additionally, baseline mindfulness levels were associated with differential intervention effects, with higher initial mindfulness related to larger positive mental health gains (*d* = 0.82 vs. *d* = 0.51, *p* < 0.01). However, the causal nature of these relationships requires further investigation.

In summary, both cross-sectional and intervention analyses provided evidence for the relationships between mindfulness, mental health dimensions, and sleep quality. The structural equation modeling supported the dual-factor framework, while the intervention results demonstrated MBSR’s effects on both positive and negative mental health outcomes. Sleep quality emerged as a potentially important factor, though its precise role requires further investigation. These findings provide a foundation for examining the theoretical and practical implications of MBSR in university settings.

## 4 Discussion

### 4.1 MBSR’S effects through a dual-factor lens

The application of the dual-factor model to MBSR research revealed several important insights. First, our findings supported the model’s basic premise of distinct but related positive and negative mental health dimensions, with the structural model accounting for 53% of variance in outcomes. This aligns with Johnson et al.’s (2024) findings in general student populations, but extends their work by demonstrating the model’s utility specifically in mindfulness intervention research.

However, our results also suggest some nuances not previously emphasized in dual-factor literature. While studies like Cheng et al. (2024) typically treat positive and negative dimensions as parallel constructs, our intervention data revealed distinct temporal patterns: negative symptom reduction showed more immediate effects, while positive mental health improvements emerged more gradually but showed greater stability. This temporal distinction adds a new dimension to the dual-factor model’s application in intervention research.

Furthermore, our findings provide important nuances to previous MBSR research. While sharing some commonalities with Conley et al.’s (2024) work regarding positive mental health outcomes (*d* = 0.71), our results revealed more complex patterns in negative symptom reduction. Both intervention and control groups showed improvements in psychological distress over time, suggesting that multiple factors may contribute to symptom reduction in university settings. This finding extends beyond previous research by highlighting how mindfulness interventions may operate differently across the dual-factor spectrum, potentially working in concert with natural recovery processes and existing support systems. These nuanced findings likely reflect our more comprehensive measurement approach and careful consideration of both between-and within-group changes in university student populations.

These findings have important implications for both theory and practice. From a theoretical perspective, our results extend the dual-factor model by demonstrating how interventions might differentially affect positive and negative dimensions over time. This temporal aspect has not been extensively explored in previous dual-factor research (Johnson et al., 2024; Cheng et al., 2024), suggesting a new direction for understanding how mental health interventions operate.

From a practical perspective, our findings support MBSR’s potential as a comprehensive mental health intervention in university settings. The moderate to large effect sizes across both dimensions suggest that group-based MBSR could effectively supplement traditional one-on-one services, particularly valuable given that counseling centers typically serve only 10–15% of the student population. However, the differential response patterns we observed suggest that practitioners might need to adjust their expectations and outcome assessments based on which dimension of mental health they are targeting.

The structural relationships revealed in our cross-sectional analyses provide additional context for these intervention effects. The balanced associations between mindfulness and both positive (*β* = 0.38) and negative (*β* = −0.31) dimensions align with theoretical predictions about mindfulness’s broad impact on mental health. However, our finding of distinct pathways for positive and negative outcomes suggests that the mechanisms through which mindfulness affects mental health may be more complex than previously understood. This complexity aligns with recent theoretical developments (LaMontagne et al., 2024) but adds important empirical support specifically in the context of university student mental health.

### 4.2 Mechanisms underlying mental health improvements

Our investigation revealed two key mechanisms underlying MBSR’s effects on mental health outcomes. First, sleep quality emerged as a mediator between mindfulness practice and mental health outcomes, particularly for positive mental health (indirect effect = 0.14, 95% CI [0.08, 0.21], *p* < 0.01), remaining robust even when controlling for other potential mechanisms (*β* = 0.12, 95% CI [0.06, 0.18]). Second, initial mindfulness levels moderated intervention effectiveness, with higher baseline mindfulness showing more rapid improvements in positive dimensions (*β* = 0.34, *p* < 0.01). These findings identify specific pathways through which mindfulness practice enhances mental health outcomes.

Neuroimaging research suggests that mindfulness training modulates activity in brain networks involved in attention regulation and emotional processing. These neural mechanisms may underlie the observed relationships between mindfulness practice and psychological outcomes, particularly through enhanced attention control and emotional regulation. Furthermore, mindfulness-related changes in parasympathetic activation may explain its association with sleep quality through reduced pre-sleep arousal, providing a neurobiological basis for understanding the complex relationships we observed between mindfulness practice, sleep quality, and psychological functioning.

Supporting these neurobiological mechanisms, our cross-sectional analyses indicated significant associations between sleep quality and mental health outcomes (*r* = −0.306 to −0.247, *p* < 0.01), a pattern consistent with both traditional mindfulness theory (Baer, 2003) and recent empirical work by Lai et al. (2024) on college students’ psychological well-being.

The intervention phase provided additional insights into these relationships, revealing patterns that align with contemporary theoretical frameworks of mindfulness and sleep regulation. Observed changes in sleep quality coincided with changes in mental health outcomes (*η*^2^ = 0.075, *p* = 0.004), though the patterns were more nuanced than previously reported. While Sun et al. (2024) observed predominantly unidirectional associations between sleep quality and symptom reduction, our dual-factor approach revealed more complex bidirectional relationships across both positive and negative mental health dimensions. These findings extend previous work by Zheng et al. (2024) by suggesting multiple pathways through which mindfulness practice may relate to psychological functioning.

Recent theoretical work by Johnson et al. (2024) and Conley et al. (2024) suggests that mindfulness interventions likely operate through multiple complementary mechanisms. Our findings support this proposition while adding important nuances. The temporal patterns we observed – with some mechanisms showing immediate effects and others emerging more gradually – suggest that the pathways through which MBSR influences mental health may be more dynamic than previously understood. However, the precise nature of these mechanisms and their interactions requires further investigation, particularly in educational settings where contextual factors might influence intervention processes.

These findings contribute to ongoing debates about mechanism specificity in mindfulness interventions. This debate centers on whether mindfulness interventions operate through universal or differentiated mechanisms. Some researchers argue for uniform mechanisms, suggesting that mindfulness primarily works through enhanced attention and awareness across all outcomes (Johnson et al., 2024; Conley et al., 2024). Others propose more differentiated models, pointing to evidence that different outcomes might be achieved through distinct pathways (LaMontagne et al., 2024). This debate has important implications for both theory development and intervention design.

Our results offer several insights into this controversy. While we found some evidence of common mechanisms affecting both positive and negative outcomes, our data more strongly supports a differentiated model. This aligns with LaMontagne et al.’s (2024) “differential response model” but extends it in important ways. Our findings suggest that mechanisms’ effectiveness varies along three dimensions: individual characteristics, temporal progression (immediate versus gradual effects), and outcome type (positive versus negative mental health). This three-dimensional variation advances the mechanism specificity debate by considering not just what mechanisms are involved, but when and for whom they are most effective.

This perspective helps reconcile seemingly contradictory findings in previous research. For instance, while Wei et al. (2024) reported uniform mechanism effects in their short-term study, and Zheng et al. (2024) found differentiated effects in their longitudinal work, our results suggest both might be valid but at different time points. Similarly, our findings help explain why some studies find strong sleep quality effects (Lai et al., 2024) while others report minimal impact (Sun et al., 2024) – the difference might lie in when and in which populations these effects were measured.

The role of individual differences emerged as particularly noteworthy in our analyses. Baseline mindfulness levels significantly moderated treatment effects (interaction *β* = 0.24, *p* < 0.01), while initial sleep quality showed moderating effects on the mindfulness-mental health relationship (interaction *β* = 0.19, *p* < 0.01). These findings both align with and extend previous work by Cowand et al. (2024), who found individual differences in intervention response but did not examine specific moderating factors. The identification of these moderators suggests potential ways to optimize intervention effectiveness through more targeted approaches.

Several limitations in current understanding of these mechanisms warrant attention. First, while our cross-sectional data suggested sleep quality as a potential mediating pathway, the intervention phase revealed more complex temporal patterns than previously reported. Second, the relationship between sleep quality and mental health outcomes might be bidirectional, a possibility our current design could not fully address. Third, the relative importance of different mechanisms might vary across cultural and educational contexts, requiring further investigation in diverse settings.

Future research should prioritize several key areas to advance our understanding of these mechanisms. First, longitudinal studies with more frequent assessment points could better capture the dynamic nature of mechanism activation. Second, person-centered analyses might help identify subgroups who respond differently to specific mechanisms. Third, cross-cultural studies could examine how these mechanisms operate in different educational contexts. Such investigations would contribute to developing more nuanced and effective approaches to mindfulness-based interventions in university settings.

### 4.3 Temporal dynamics and implementation considerations

Our longitudinal data revealed distinct temporal patterns in participants’ responses to MBSR participation. Consistent with contemporary mindfulness theory (Kabat-Zinn, 2013), we observed differential patterns across outcome domains: changes in negative symptoms showed gradual attenuation over 6 months (reduction from *d* = 0.62 to *d* = 0.41), while positive mental health measures maintained relative stability (*d* = 0.58 at follow-up). These patterns align with theoretical frameworks suggesting that different aspects of psychological functioning may follow distinct developmental trajectories (Lucas-Thompson et al., 2023).

Analysis of participation patterns provided insights into engagement with mindfulness practices. Participants’ mindfulness practice logs showed associations between regular practice and subsequent outcomes (*r* = 0.42, *p* < 0.01 at 6-month follow-up). Additionally, early changes in sleep quality patterns (observed in 37% of participants by week 4) showed relationships with later psychological functioning (*β* = −0.31, *p* < 0.01). These observations align with theoretical perspectives on the gradual development of mindfulness skills (Baer, 2003) and suggest the importance of sustained practice.

Further analysis of individual response patterns revealed important baseline characteristics that influenced intervention outcomes. Participants with higher initial mindfulness levels (top quartile) showed more rapid improvements in positive dimensions (*β* = 0.34, *p* < 0.01), while those with poorer baseline sleep quality demonstrated stronger improvements in negative symptoms (*β* = −0.29, *p* < 0.01). These findings suggest that initial assessment of these characteristics might help identify participants who could benefit most from specific aspects of the intervention.

Analysis of temporal patterns also revealed the potential influence of external factors on both groups’ outcomes. The academic calendar and examination cycles inherent to university settings may have affected students’ psychological states across the study period. Additionally, the availability of general mental health support services and increased mental health awareness on campus could have benefited all participants regardless of group assignment. The process of regular psychological assessment itself may have enhanced self-awareness among all participants. These contextual influences align with contemporary understanding of how educational environments can shape intervention outcomes, suggesting the importance of considering broader institutional and temporal factors when implementing mindfulness programs in university settings.

Practice engagement patterns provided additional insights into the relationships between mindfulness training and psychological outcomes. Analysis of practice logs revealed associations between consistent participation (≥4 sessions/week) and outcome maintenance for both positive (*d* = 0.67 vs. *d* = 0.41, *p* < 0.01) and negative (*d* = 0.58 vs. *d* = 0.33, *p* < 0.01) dimensions at follow-up. These observations align with both traditional mindfulness theory (Kabat-Zinn, 2013) and contemporary research on skill acquisition in contemplative practices, suggesting the potential value of sustained engagement with mindfulness practices.

The observed implementation patterns suggest considerations for MBSR program delivery in educational settings. Drawing on both empirical observations and theoretical frameworks, our findings point to several aspects that may warrant attention in future research and practice. The differential patterns in outcome stability align with contemporary understanding of psychological change processes, suggesting potential value in considering both immediate and longer-term relationships between practice engagement and psychological functioning. These observations contribute to ongoing discussions about optimizing mindfulness-based approaches in educational settings (Deroche et al., 2025), while acknowledging the need for continued investigation of individual differences and contextual factors.

The temporal and relationship patterns observed in our study offer relevant considerations for educational practice. Our analyses suggest associations between sleep quality and psychological outcomes (*η*^2^ = 0.075, *p* = 0.004), a finding that may be particularly relevant in educational settings where sleep patterns have been associated with students’ psychological functioning (Lai et al., 2024). These observations complement recent research suggesting relationships between sleep patterns and various aspects of student well-being and academic experiences (Sun et al., 2024). The different patterns we observed across positive and negative dimensions add nuance to LaMontagne et al.’s (2024) discussion of mental health support in academic settings.

Our observations also suggest possible approaches for integrating mindfulness practices within existing student support frameworks. The observed patterns of practice engagement and outcome maintenance (*d* = 0.67 vs. *d* = 0.41 for positive outcomes) align with Conley et al.’s (2024) perspectives on sustained mindfulness practice. These findings complement Wu and Qin’s (2025) research on the potential benefits of incorporating mindfulness approaches within established student support systems. These observations may inform future efforts to implement mindfulness-based programs in educational settings, while considering practical aspects of academic schedules and resources.

### 4.4 Methodological contributions to educational psychology research

Our integrated methodological approach offers several contributions to educational psychology research methods. The sequential mixed-methods design—combining cross-sectional analysis (*N* = 406) with a randomized trial (*N* = 120)—demonstrated practical advantages for studying psychological interventions in educational settings. This approach provided sufficient statistical power at both phases (>0.90 for cross-sectional analyses and 0.85 for intervention effects), while revealing patterns that single-method studies have typically missed. For instance, while our cross-sectional data showed balanced associations between mindfulness and both positive and negative outcomes (*β*s = 0.38 and −0.31), the experimental phase revealed distinct temporal trajectories in these relationships, with positive effects showing greater stability over time (6-month test–retest *r* = 0.68 vs. *r* = 0.43 for negative effects). This temporal dimension is particularly important in educational research, where interventions must be evaluated within the natural cycles of academic terms.

In educational psychology research, where both individual development and group-level interventions must be considered, our approach demonstrates particular value. The sequential design allowed us to first establish the structural relationships among variables in a large sample before testing causal mechanisms in the intervention phase. This progression aligns with best practices in educational intervention research (Villalón et al., 2023), where understanding the theoretical relationships should precede intervention testing. Moreover, our approach addressed a common limitation in educational psychology studies—the trade-off between statistical power and depth of assessment—by providing adequate power at both the model-testing phase (*N* = 406) and the intervention phase (*N* = 120).

The longitudinal component of our design revealed important temporal patterns that have specific relevance for educational research. The differential stability of positive versus negative outcomes (*r* = 0.68 vs. *r* = 0.43 at 6-month follow-up) suggests that intervention effects in educational settings may follow different trajectories depending on the outcome measured. This finding extends recent work on the temporal dynamics of educational interventions (Lucas-Thompson et al., 2023), highlighting the importance of appropriate follow-up periods in academic research. Furthermore, our approach to monitoring implementation factors (practice consistency, early response patterns) provides a methodological template for evaluating similar interventions in educational contexts, where fidelity and engagement are crucial concerns.

Our sequential mixed-methods approach specifically enhanced mediation analysis in educational intervention research. By examining sleep quality’s mediating role in both cross-sectional and experimental phases, we identified temporal variations that would be missed in single-method designs. Cross-sectional analysis identified sleep quality as a significant mediator (*β* = 0.14, 95% CI [0.09, 0.19]), while experimental data revealed that this pathway’s strength varied across intervention phases. This temporal dimension of mediation is particularly relevant for educational interventions, where the timing of effects may interact with academic schedules and pressures. As Conley et al. (2024) note, understanding not just if but when intervention mechanisms operate is crucial for optimizing implementation in educational settings.

Our longitudinal measurement analyses revealed specific psychometric challenges relevant to educational assessment. The differential pattern of measurement invariance—with mindfulness measures maintaining stability (CFI > 0.95, RMSEA < 0.06) while mental health measures showed structural shifts (ΔCFI = 0.02–0.03)—highlights an important consideration for evaluating psychological interventions in educational settings. This finding has important implications for educational assessment, suggesting that students’ conceptualization of well-being may evolve as they engage with intervention content. This aligns with research by Johnson et al. (2024) on how mindfulness practice influences psychological constructs in educational settings. Such measurement considerations are crucial when evaluating educational interventions that aim to change not just behaviors but underlying psychological constructs.

The integration of multiple analytical approaches in our study contributes to ongoing methodological discussions in educational psychology research regarding the examination of both group-level patterns and individual variations. Cross-lagged panel analyses suggested bidirectional associations between mindfulness practice and sleep quality patterns (practice-sleep: *β* = 0.24, *p* < 0.01; sleep-practice: *β* = 0.19, *p* < 0.01), offering insights into the complex relationships between practice engagement and sleep-related experiences. Growth mixture modeling revealed varied trajectories in psychological functioning (linear: 45%, quadratic: 38%, delayed: 17%), patterns that complement current perspectives on individual differences in educational interventions (Wu and Qin, 2025). These observations support considerations of both person-centered and variable-centered analytical approaches in educational research.

These methodological insights suggest three specific directions for future educational psychology research: First, researchers should consider the temporal dynamics of intervention effects, particularly in relation to academic cycles. Our finding that positive and negative outcomes follow different temporal trajectories suggests that evaluation time points should be carefully selected to capture the full spectrum of intervention effects. Second, mixed-method designs that combine structural modeling with experimental approaches offer unique advantages for educational research, where both theoretical understanding and practical effectiveness must be addressed. Finally, measurement approaches in educational intervention research should account for potential shifts in how students conceptualize psychological constructs as they engage with intervention content. These recommendations align with recent calls for methodological innovation in educational psychology (LaMontagne et al., 2024) and offer practical strategies for advancing research in this field.

### 4.5 Study limitations and methodological considerations

Several methodological limitations warrant careful consideration when interpreting our findings. First, the single-site sampling from one university limits generalizability to other educational and cultural contexts. Different institutional environments, cultural backgrounds, and educational systems may influence how students engage with and respond to mindfulness interventions. Second, while our sample size (*N* = 120) provided adequate power for primary hypotheses, it limited our ability to conduct more fine-grained subgroup analyses across diverse student characteristics.

Third, our reliance on self-report measures, while standard in psychological research, introduces potential measurement biases. Particularly relevant is social desirability bias, where participants might report changes they believe are expected or desired. This concern is especially pertinent in educational settings where students may feel implicit pressure to demonstrate improvement. The use of self-report measures also means our findings reflect perceived rather than objective changes in mindfulness and psychological functioning.

Fourth, although our six-month follow-up captured important temporal patterns, educational interventions may have longer-term effects that continue developing throughout students’ academic careers. Future research would benefit from multiple-site sampling, incorporation of objective measures, and longer follow-up periods to address these limitations.

### 4.6 Future directions for educational research

Building on our findings and acknowledging the limitations, we identify three priority areas for advancing mindfulness research in educational settings. First, future studies should examine how mindfulness interventions operate within diverse educational contexts. This includes investigating how institutional characteristics (e.g., academic calendars, support resources) and student populations (e.g., undergraduate vs. graduate, different disciplines) moderate intervention effectiveness. As Wu and Qin (2025) have demonstrated, the educational context significantly influences how mindfulness interventions are received and implemented.

Second, researchers should further explore the temporal dynamics of mindfulness effects in relation to academic cycles. Our finding that positive and negative outcomes follow different trajectories suggests the need for more sophisticated longitudinal designs that align with natural educational rhythms. This might include intensive assessment during high-stress academic periods (e.g., examinations) and longer follow-up during academic transitions. Such designs would address Lucas-Thompson et al.'s (2023) call for more ecologically valid approaches to studying mindfulness in educational settings.

Third, greater attention should be paid to the practical implementation of mindfulness interventions within existing educational structures. Our findings on the importance of practice consistency suggest that research should examine how mindfulness programs can be effectively integrated into academic schedules and student life. This integration is particularly important given the competing demands on students’ time and attention (LaMontagne et al., 2024). Collaborative research between education specialists and mindfulness researchers offers promising opportunities to develop interventions that are both theoretically sound and practically feasible in educational environments.

## 5 Conclusion

By integrating cross-sectional and experimental evidence, this study enriches our understanding of MBSR’s role in educational settings through a dual-factor model framework. The findings reveal distinct patterns of intervention effects, with particularly robust and sustained improvements in positive mental health (*d* = 0.71), and more complex trends observed in psychological distress reduction. These results underscore the importance of situating mindfulness interventions within the broader university context and highlight their potential as a targeted and scalable approach to student mental health promotion.

The study makes several contributions to educational research and practice. First, our dual-factor approach reveals how mindfulness interventions may work differently across mental health dimensions, with patterns that reflect the complex nature of psychological well-being in academic settings. Second, the findings suggest that MBSR may function most effectively when integrated with existing support systems, pointing to the importance of comprehensive, multi-faceted approaches to student mental health. Third, our sequential mixed-methods design provides valuable insights into both the broader patterns of relationships and specific intervention effects, offering methodological guidance for future research in educational settings.

While acknowledging the limitations of our sample and follow-up duration, this study advances our understanding of how mindfulness-based interventions function within educational contexts. The findings suggest that MBSR could serve as a valuable component in a comprehensive approach to university mental health services, particularly in enhancing positive mental health outcomes. Future research should continue to examine how mindfulness interventions interact with existing support systems, focusing on understanding individual response patterns, temporal dynamics, and the various pathways through which such interventions may contribute to student well-being. Such investigations would help develop more nuanced, evidence-based approaches to supporting student mental health that recognize both direct intervention effects and the broader context of academic support systems.

## Data Availability

The raw data supporting the conclusions of this article will be made available by the authors, without undue reservation.
